# Case Report of Unusual Facial Swelling in an 8-Month-Old

**DOI:** 10.21980/J8M06F

**Published:** 2021-07-15

**Authors:** Amanda E Mulcrone, Zobiya Z Momin, Corrie E Chumpitazi

**Affiliations:** * Baylor College of Medicine, Department of Pediatrics, Section of Pediatric Emergency Medicine, Houston, TX; ^ Baylor College of Medicine, Department of Pediatrics, Houston, Tx

## Abstract

Rhabdomyolysis is a potentially life-threatening disease defined by the release of intracellular contents into the body’s circulation after muscle damage. It is most commonly seen in adult patients who present with crush injuries, overexertion, or prolonged immobility due to drug and/or alcohol overdose. Fortunately, it is rare in young children. We present the case of an 8-month-old girl with progressively worsening right-sided facial swelling that was associated with significant drooling and intraoral edema. The patient had been found lying prone and wedged between a mattress and the adjacent bedroom wall. After laboratory and imaging work-up, this child’s injury was the result of prolonged facial muscle compression with resultant rhabdomyolysis. The patient improved during hospital admission with intravenous (IV) fluids, two days of steroids for facial swelling, and five days of antibiotics to treat superficial cellulitis. At 2-week follow-up, her swelling resolved with minimal residual facial scarring and a mild right facial nerve palsy. This patient’s unusual history and clinical presentation challenges providers to explore rhabdomyolysis as a differential diagnosis in young children who present with localized severe swelling.

**Topics:**

Facial edema, rhabdomyolysis, facial cellulitis, non-accidental trauma.

**Figure f1-jetem-6-3-v18:**
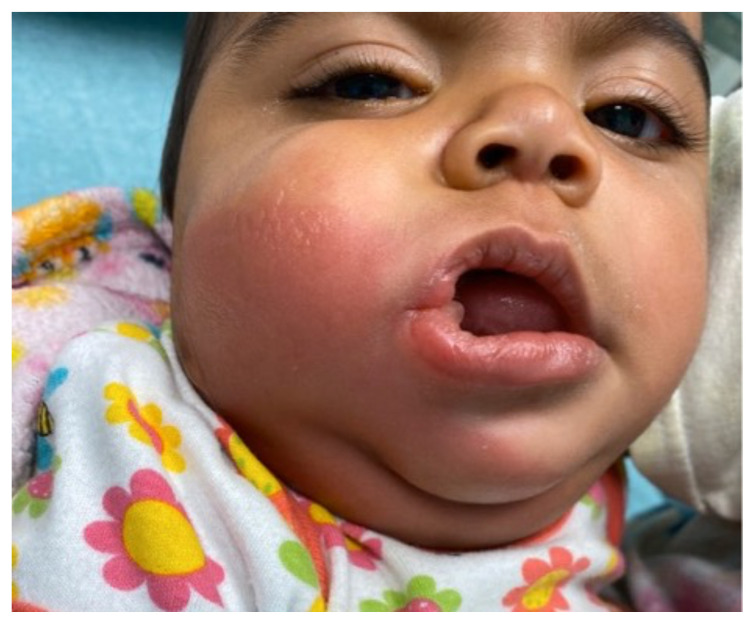


**Figure f2-jetem-6-3-v18:**
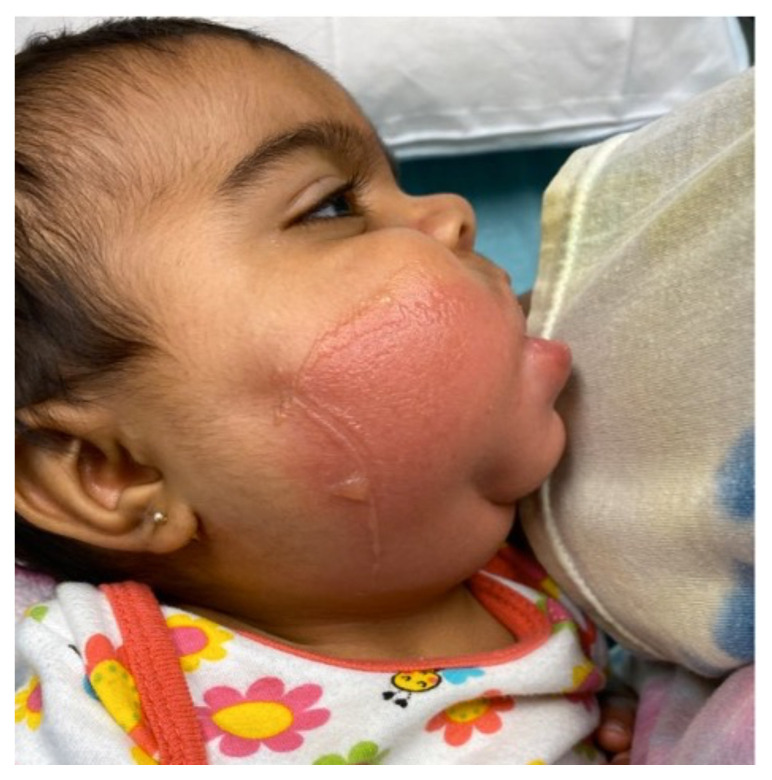


**Figure f3-jetem-6-3-v18:**
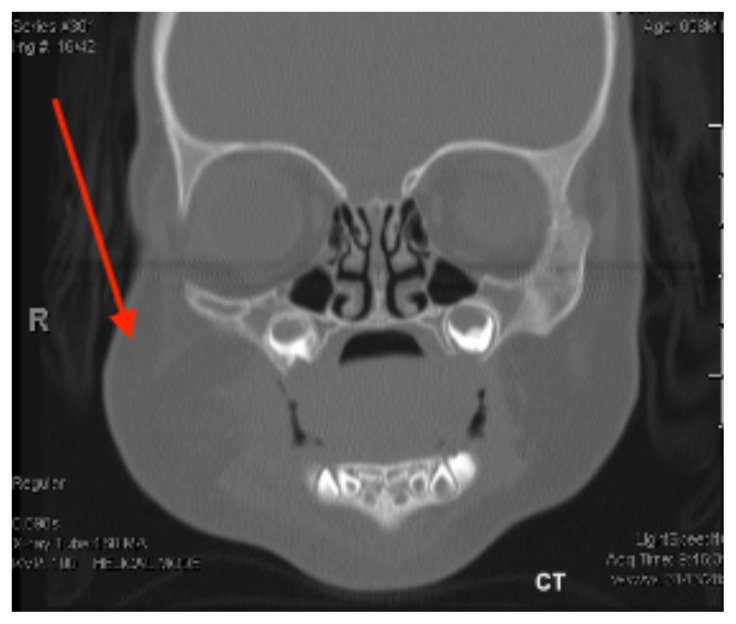


**Figure f4-jetem-6-3-v18:**
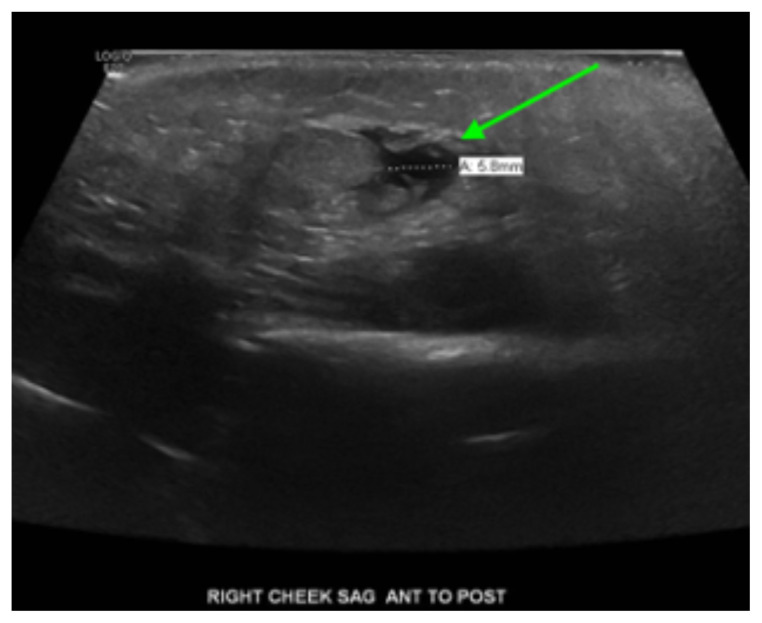


## Brief introduction

[Fig f1-jetem-6-3-v18][Fig f2-jetem-6-3-v18][Fig f3-jetem-6-3-v18][Fig f4-jetem-6-3-v18]Rhabdomyolysis is a disease in which intracellular contents are released into circulation after muscle damage and can lead to many life-threatening complications. The most common causes of pediatric rhabdomyolysis include viral infections, traumatic injury, exercise, medications, ingestions, and genetic disorders.^1^,^2^ While the exact incidence of rhabdomyolysis in pediatric patients is unknown, there are approximately 25,000 cases of rhabdomyolysis reported annually in both children and adults in the United States.^1^

Identifying rhabdomyolysis can be challenging in the young pediatric patient who is non-verbal. While the classic presentation of rhabdomyolysis includes a triad of myalgias, muscle weakness, and dark urine, it is common for a pediatric patient’s only presenting symptom to be myalgias.^1^ Laboratory testing is needed for prompt diagnosis and initiation of medical therapies to prevent significant complications including electrolyte abnormalities, acute kidney injury, and disseminated intravascular coagulation (DIC).^1^–^3^ In this case, we discuss the unusual presentation of an infant with severe facial swelling with an evaluation that supports the diagnosis of rhabdomyolysis in the emergency setting.

## Presenting concerns and clinical findings

A previously healthy 8-month-old girl presented to the emergency department with progressively worsening right-sided facial swelling. The patient was last seen in her usual state of health at bedtime, when the mother placed her in the middle of a twin mattress. The patient does not have a crib. Approximately 8 hours later, the mother reported she found the patient lying prone with her body wedged between the mattress and the adjacent bedroom wall. The right side of the patient’s face was directly against the corner of the mattress. Mother presumes that the patient rolled towards the wall during the night.

Upon arrival to the emergency department, the patient was afebrile with normal vital signs. The patient was drooling and refused to drink her bottle. She was resting calmly in her mother’s arms without stridor or respiratory distress. The patient had significant right cheek warmth and swelling, intraoral edema, and tongue swelling. The superficial skin was erythematous with blistering. The affected area was painful to touch. The degree of the patient’s facial swelling and erythema can be appreciated by viewing the included patient photographs.

## Significant findings

Initial laboratory evaluation demonstrated a leukocytosis to 19 (normal 5.98 – 13.51 × 10*3/uL) with left shift and elevated creatine kinase (CK) to 12,249 (normal 60–305 U/L). The patient also had an elevated aspartate transaminase (AST) of 320 (normal 15–50 U/L) and amylase of 115 (normal 30–115 U/L). Other labs, including alanine transaminase (ALT), lipase, and creatinine were normal.

Head and maxillofacial computed tomography (CT) showed right-sided cheek, submandibular, and submental soft tissue swelling (red arrow), without skull or facial fracture, intracranial hemorrhage, or abscess.

Facial ultrasound revealed local inflammatory changes such as increased echogenicity and heterogeneity in the soft tissues of the right cheek, suggestive of soft tissue edema. There was evidence of a prominent right parotid gland with increased heterogeneity suggestive of a traumatic injury. Additionally, facial ultrasound demonstrated a 6mm ill-defined anechoic collection within the right cheek without increased doppler flow (green arrow), thought to represent a focal area of edema instead of an abscess.

## Patient course

Initial differential diagnoses in the emergency department included infection, non-accidental trauma (NAT), burn, or pressure wound. Elevations of the AST and amylase in combination with the consideration of NAT resulted in a CT of the abdomen/pelvis being obtained and ruling out an intra-abdominal injury. Due to unusual history and severe presentation, a child protection team consult was obtained and skeletal survey radiographs were unremarkable.

The patient was admitted to the hospital. Her injuries were deemed plausible by reported history by the child protection team. The patient’s CK level down trended with continuous IV fluids and she maintained normal electrolytes, including creatinine. Given concern for progression to airway compromise due to the patient’s extensive facial and intraoral swelling, she was given dexamethasone for the first 48 hours of her hospitalization which improved the swelling. The patient was started on a five-day course of clindamycin to cover skin flora for overlying cellulitis which improved the localized erythema. At 2-week follow-up, her facial swelling resolved with minimal residual facial scarring and mild right facial nerve palsy.

## Discussion

This injury was caused by prolonged facial muscle compression resulting in rhabdomyolysis. Rhabdomyolysis is defined by the release of intracellular contents into the body’s circulation after muscle damage.^1^ Trauma with a direct injury to the affected muscle is one of the most common causes of rhabdomyolysis in pediatric patients.^1^,^2^ Skeletal muscle can break down after as little as one hour of pressure and immobility, causing muscle cell death and leading to release of intracellular components into circulation.^1^,^4^ Intracellular components that are released into circulation include excess potassium, calcium, phosphorus, uric acid, and muscle enzymes such as creatine kinase (CK) and aspartate transaminase (AST).^1^ CK levels are often trended due to correlation as the most sensitive enzyme marker for muscle breakdown.^1^ Most content experts consider a CK level more than 5 times the upper limit of normal diagnostic level as a marker for pediatric rhabdomyolysis, but there is no set diagnostic criteria for other laboratory values including AST.^1^

Rhabdomyolysis typically presents with the clinical triad of myalgias, muscle weakness, and dark colored urine; however, facial muscles are rarely involved.^1^,^3^ Rhabdomyolysis can lead to life-threatening complications including significant electrolyte imbalance, acute renal failure, and disseminated intravascular coagulation; thus it is an important illness to recognize.^1^–^3^ Consideration of rhabdomyolysis can be challenging in the non-verbal infant, especially in the case of our patient’s unique and unusual presentation of localized severe facial swelling.

In the adult population, cases of facial rhabdomyolysis due to drug toxicity and overdose have been reported.^2^,^5^ A 23-year-old was noted to have right facial rhabdomyolysis after being found comatose lying face down for 7–12 hours in the setting of elevated alcohol and cocaine levels.^2^ Imaging is not required to diagnose rhabdomyolysis but may aid in reaching the appropriate diagnosis in cases that are not clearly evident.^2^ In the previously described 23-yer-old, head and neck CT imaging revealed “swelling of the right masseter muscle and parotid gland with associated soft tissue edema and fat stranding in the subcutaneous tissue.”^2^ These findings of soft tissue edema were similarly reported on our patient’s imaging as well. There is limited data regarding ultrasound findings in rhabdomyolysis; however, it has been reported in prior cases of rhabdomyolysis that ultrasound imaging demonstrated “edematous hypoechoic muscle containing multiple hyperechoic foci with disorganized fascicular architecture.”^6^ In our patient, only inflammatory changes were reported on ultrasound imaging.

Facial rhabdomyolysis should be treated as aggressively as rhabdomyolysis of any other muscle. Aggressive volume replacement is essential to prevent acute kidney injury during progression of muscle breakdown. Additionally, it is crucial to monitor electrolyte imbalance, particularly hyperkalemia, and the continuing effects on renal function such as urine output.^2^ Our patient appropriately responded to intravenous volume replacement and the CK level, along with AST, slowly normalized during the hospitalization. Our patient never developed an acute kidney injury.

Additionally, our patient was found to have a right facial nerve palsy on a follow-up appointment two weeks after hospital discharge. Extensive external pressure and inflammation onto the superficial branches of the facial nerve can compromise blood flow leading to local ischemia and decreased transmission of action potentials, resulting in nerve malfunction.^7^ This nerve palsy, however, resolves as inflammation improves.^7^

Finally, the unusual history and presentation of this patient raised concern for possible NAT. Child abuse is a significant cause of pediatric morbidity and mortality, so identifying abused children with suspicious injuries and early recognition of abuse can be lifesaving.^8^ NAT is unfortunately common, with over 2 million reports of suspected child maltreatment being investigated annually in the United States.^8^ It is estimated there are over 1500 annual child deaths attributed to abuse, with over 80% of these deaths occurring in young children under the age of four.^8^

Identifying child abuse is not always simple and there are many disease processes that can be mistaken for child abuse. Unusual accidents resulting in atypical injuries do happen to children, but NAT should be included in the differential for any young patient with unusual injuries. In general, examination findings that can be suggestive of abuse include mouth injuries in a pre-ambulatory infant and injuries to nonbony locations including the ears or face, as was the case in our patient.^8^

When severe or multiple injuries are present in young children, it is recommended to screen for abdominal trauma with laboratory tests, including the levels of serum liver enzymes (ALT and AST) and the pancreatic enzymes (amylase and lipase).^9^,^10^ There have been many studies reviewing intra-abdominal trauma from child abuse and abnormal levels of liver and pancreatic serum enzymes.^11^–^13^ Although abnormal levels of liver and pancreatic enzymes are not diagnostic of detectable injury, abnormally elevated levels do indicate a sign for further investigation.^11^ It has been found that elevated hepatic transaminases increase sensitivity for finding intra-abdominal injury compared to the physical examination alone of children with concern for physical abuse.^12^ ALT and AST values greater than twice the normal limit (>90 U/L, >120 U/L respectively) are significantly associated with a positive CT finding of abdominal injury.^13^

In this patient’s emergency department course, screening liver and pancreatic enzyme labs were performed, indicating elevated AST and amylase levels. These lab elevations did lead the clinical team to perform abdominal and pelvic CT imaging to evaluate for intra-abdominal injuries at the request of the surgical trauma consult team. However, this patient’s initial laboratory and imaging evaluation prior to the abdominal CT was consistent with rhabdomyolysis due to a pressure injury. While child abuse and NAT should always be considered, this patient’s clinical presentation, the provided history of prolonged compression, and elevated CK and AST levels but normal ALT level, was indicative of rhabdomyolysis. More rapid consultation with the child protection team overnight could have perhaps delayed or avoided abdominal CT imaging and the additional radiation exposure associated with CT imaging in this young patient.^13^

## Supplementary Information








