# Supplementary figures and images for: Case Report of Unusual Facial Swelling in an 8-Month-Old
Source: J Educ Teach Emerg Med. 2021 Jul 15;6(3):V18–22. doi: 10.21980/J8M06F (PMC10332687; doi:10.21980/J8M06F)

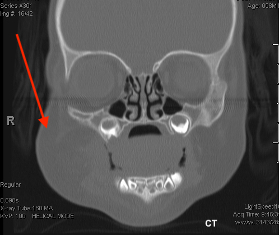

Supplement: Supplementary file 1 [file jetem-6-3-v18-supp1.jpeg]

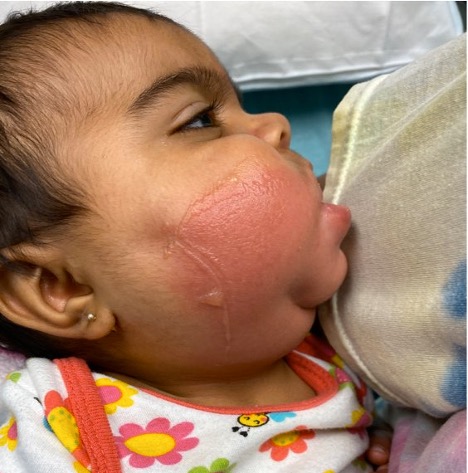

Supplement: Supplementary file 2 [file jetem-6-3-v18-supp2.jpg]

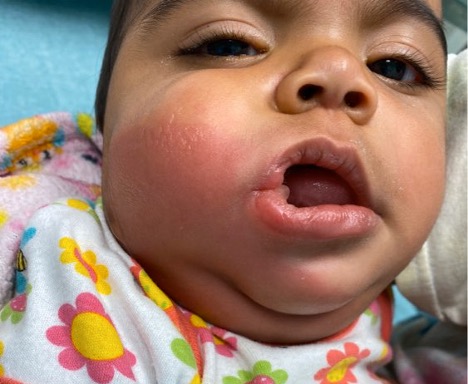

Supplement: Supplementary file 3 [file jetem-6-3-v18-supp3.jpg]

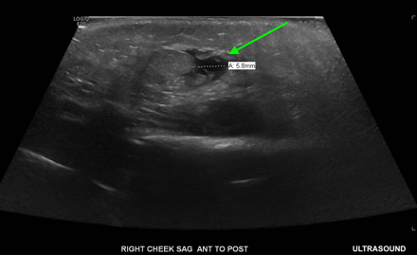

Supplement: Supplementary file 4 [file jetem-6-3-v18-supp4.jpeg]
